# Prediction of plasma efavirenz concentrations among HIV-positive patients taking efavirenz-containing combination antiretroviral therapy

**DOI:** 10.1038/s41598-017-16483-2

**Published:** 2017-11-23

**Authors:** Sung-Hsi Huang, Shu-Wen Lin, Sui-Yuan Chang, Ya-Ting Lin, Chieh Chiang, Chin-Fu Hsiao, Hsin-Yun Sun, Wen-Chun Liu, Yi-Ching Su, Chien-Ching Hung, Shan-Chwen Chang

**Affiliations:** 1 Department of Emergency Medicine, National Taiwan University Hospital Hsin-Chu Branch, Hsin-Chu, Taiwan; 2 Department of Pharmacy, National Taiwan University Hospital and National Taiwan University College of Medicine, Taipei, Taiwan; 3 Graduate Institute of Clinical Pharmacy, National Taiwan University College of Medicine, Taipei, Taiwan; 4 School of Pharmacy, National Taiwan University, Taipei, Taiwan; 5 Department of Laboratory Medicine, National Taiwan University Hospital and National Taiwan University College of Medicine, Taipei, Taiwan; 6 Department of Clinical Laboratory Sciences and Medical Biotechnology, National Taiwan University College of Medicine, Taipei, Taiwan; 7 Institute of Population Health Sciences, National Health Research Institutes, Miaoli, Taiwan; 8 Department of Internal Medicine, National Taiwan University Hospital and National Taiwan University College of Medicine, Taipei, Taiwan; 9 Department of Parasitology, National Taiwan University College of Medicine, Taipei, Taiwan; 10 Department of Medical Research, China Medical University Hospital, Taichung, Taiwan; 11 China Medical University, Taichung, Taiwan

## Abstract

We investigated the predictors of plasma mid-dose concentrations (C12) of efavirenz by enrolling 456 HIV-positive patients who had received 2 nucleos(t)ide reverse-transcriptase inhibitors plus efavirenz (600 mg daily) for 2 weeks or longer and had their CYP2B6 516G>T polymorphism and efavirenz C12 determined. The median efavirenz C12 was 2.41 mg/L (IQR, 1.93–3.14). In analysis of covariance models, patients with CYP2B6 516GT and TT genotypes compared to those with GG genotype had higher efavirenz C12 (for GT genotype, an increase by 0.976 mg/L [95%CI, 0.765–1.188], and TT genotype, 4.871 mg/L [95%CI, 4.126–5.616]), while per 10-kg increment in weight decreased C12 by 0.199 mg/L (95%CI, 0.111–0.287). Models incorporating CYP2B6 516G>T polymorphism and weight had moderate predictive values in predicting efavirenz C12 ≥ 2 mg/L (ROC area under curve = 0.706 [95%CI, 0.656–0.756]). In the absence of CYP2B6 516G>T polymorphism, weight ≤58 kg provided better predictabilities for efavirenz C12 ≥ 2 mg/L (probability, 77.1% [95%CI, 69.0–83.5%] for weight = 50 kg and 70.6% [95%CI, 64.1–76.4%] for weight = 58 kg).

## Introduction

Efavirenz (EFV), a non-nucleoside reverse-transcriptase inhibitor (nNRTI), is one of the most widely used antiretroviral agents^1^. With indications for both HIV-positive adult and pediatric populations, an extensive experience in both developed and resource-limited countries, and a well-known and manageable interaction with anti-tuberculous agents, it remains the cornerstone in the antiretroviral therapy and the universal access approach towards tackling the current HIV epidemic.

The recommended EFV dose for an HIV-positive adult patient is 600 mg daily and the suggested therapeutic range of plasma mid-dose concentration (C12) of EFV is 1 to 4 mg/L. While plasma EFV C12 lower than 1 mg/L were reportedly linked to decreased antiretroviral activity^2,3^, higher EFV C12 above 4 mg/L were linked to increased adverse effects of the central nervous system^2,4,5^, abnormal liver profile within 6 weeks^6,7^, and dyslipidemia and hyperglycemia^8^ in the long term. However, the optimal dose of EFV has been debated over the past several years.

ENCORE1 study has shown that EFV at 400 mg daily is non-inferior to the standard 600 mg, when combined with tenofovir disoproxil fumarate (TDF) and emtricitabine (FTC) in antiretroviral-naïve adult patients^9^. The pharmacokinetic investigations in this group of patients demonstrated that few patients, if any, taking 400 mg of EFV had EFV C12 below the therapeutic range^10^. Furthermore, in the phase II study comparing daily 200 mg, 400 mg or 600 mg of EFV in 137 patients, there was no significant difference among the three dosing groups in their clinical efficacy^11^. In addition, the unwanted drug effects due to high plasma concentrations may also be ameliorated by reductions of the EFV dose in areas where regimens consisting of EFV plus 2 nucleos(t)ide reverse-transcriptase inhibitors (NRTIs) remain the first-line combination antiretroviral therapy (cART).

A wide interpatient variability exists regarding the plasma EFV C12^12,13^. Previous reports have found that certain factors may be associated with this variability, including gender^12,14^, weight^15–17^, race^12,18^, concomitant medications^19^, and genetic polymorphism at specific loci encoding the cytochrome P450 enzymes^20^. Under the scheme of therapeutic drug monitoring (TDM), successful reduction of EFV to a daily dose of 300 mg or 200 mg has been reported^21,22^. However, considering that EFV in combination with TDF/FTC remains the first-line cART in areas where TDM is generally unavailable, HIV care providers may need other tools to guide dose reduction. Our previous study has shown that EFV dose reduction from 600 mg to 300 mg was associated with a 46.2% reduction in the plasma EFV C12^23^.

Accumulating evidence suggests that the minimum effective concentration of EFV may be lower than 1 mg/L^24,25^. Thus, theoretically, the EFV dose of a patient with a plasma EFV C12 of 2 mg/L or above can potentially be reduced to 300 mg without compromising its efficacy.

In the present study, we aimed to investigate the clinical factors that might influence plasma EFV C12 in a Taiwanese cohort and to identify models that might be used for predicting plasma EFV C12 greater than or equal to 2 mg/L when taking EFV at a daily dose of 600 mg.

## Results

Of the 502 HIV-positive patients receiving EFV-containing regimens during the study period, 456 patients (90.8%) were enrolled and 46 patients were excluded, including 14 whose plasma was sampled outside the time frame of 12 ± 2 hours after the last dose of EFV, 27 who were concurrently taking either rifampicin, anti-epileptics or protease inhibitors, and 5 who had documented poor drug adherence. The study population was predominantly male (94.7%), with a median age of 36.2 years, weight of 65 kg and height of 171 cm (Table 1). The median nadir CD4 lymphocyte count was 231 cells/mm^3^ and 43.5% of these patients started their antiretroviral therapy with a CD4 cell count <200 cells/mm^3^. At the time of blood sampling for determinations of plasma EFV C12, the majority of the patients (91.8%; n = 415) had either a plasma HIV RNA load <50 copies/ml or a 2-log_10_ decline from baseline in those whose treatment duration was less than 6 months. The most common genotype was CYP2B6 516GG (67.1%), followed by GT heterozygotes (31.1%); and only 8 patients (1.8%) were TT homozygotes.

**Table 1:** Clinical characteristics of 456 HIV-positive patients who received efavirenz-containing combination antiretroviral therapy.

	All patient (N = 456)	Plasma efavirenz concentration <2 mg/L (N = 130)	Plasma efavirenz concentration ≥2 mg/L (N = 326)	p-value
Age, median (IQR), years	36.2 (29.4, 43.4)	34.3 (29.1, 41.5)	37.0 (29.5, 43.6)	0.1031
Male sex, n (%)	432 (94.7)	127 (97.7)	305 (93.6)	0.1020
Body weight, median (IQR), kg	65 (58, 71.8)	68 (62, 75)	64 (57, 70)	<0.0001
Body height, median (IQR), cm	171 (168, 175)	173 (170, 176)	170 (167, 174)	0.0003
Body surface area, median (IQR), m^2^	1.75 (1.66, 1.86)	1.80 (1.71, 1.90)	1.74 (1.63, 1.84)	<0.0001
Body-mass index, median (IQR), kg/m^2^	22.1 (20.3, 24.1)	22.9 (20.8, 25.1)	22.0 (20.1, 23.8)	0.0011
Chronic HBV infection, n (%)	75 (16.5)	12 (9.2)	63 (19.3)	0.0078
Chronic HCV infection, n (%)	30 (6.6)	8 (6.2)	22 (6.7)	>0.9999
Elevated aminotransferase, n (%)	75 (16.5)	19 (14.6)	56 (17.2)	0.5767
Estimated glomerular filtration rate, median (IQR), ml/min/1.73 m^2^	103.9 (91.5, 115.6)	104.6 (94.6, 118.3)	103.3 (90.9, 115.0)	0.1754
Estimated glomerular filtration rate <90 ml/min/1.73 m^2^, n (%) (N = 455)	102 (22.4)	27 (20.8)	75 (23.1)	0.6211
Nadir CD4, median (IQR), cells/mm^3^ (N = 441)	231 (78, 372)	302 (157, 460)	206.5 (61, 342)	<0.0001
Nadir CD4 <200 cells/mm^3^, n (%) (N = 441)	192 (43.5)	38 (29.9)	154 (49.0)	0.0003
PVL before initiation of cART, median (IQR), log_10_ copies/ml (N = 424)	4.87 (4.45, 5.44)	4.70 (4.32, 5.32)	4.94 (4.52, 5.46)	0.0296
CD4 at the time of sampling for plasma efavirenz concentrations, median (IQR), cells/mm^3^ (N = 450)	502 (358, 662)	531.5 (404, 704.5)	486.5 (334, 658)	0.0184
PVL at the time of sampling for plasma efavirenz concentrations, median (IQR), log_10_ copies/ml (N = 450)	1.59 (1.59, 1.72)	1.59 (1.59, 1.69)	1.59 (1.59, 1.73)	0.9436
CART duration at the time of sampling for plasma efavirenz concentrations, n (%)				0.1293
≥180 days	304 (66.7)	79 (60.8)	225 (69.0)	
30 to 179 days	111 (24.3)	40 (30.8)	71 (21.8)	
<30 days	41 (9.0)	11 (8.5)	30 (9.2)	
PVL <50 copies/ml at the time of sampling for plasma efavirenz concentrations, n (%) (N = 304)*	287 (94.4)	77 (97.5)	210 (93.3)	0.2551
PVL <50 copies/ml or 2 log_10_ decline from baseline to the time of sampling for plasma efavirenz concentrations, n (%) (N = 111)**	94 (84.7)	36 (90.0)	58 (81.7)	0.3747
CART-responsive, n (%) (N = 415)***	381 (91.8)	113 (95.0)	268 (90.5)	0.1916
Duration on cART, median (IQR), days	498.5 (78, 2246)	411.5 (57, 1965)	536 (117, 2290)	0.2103
On EFV as the first-line treatment, n (%)	388 (85.1)	112 (86.2)	276 (84.7)	0.7716
Duration on EFV, median (IQR), days	473.5 (70, 1982)	368 (50, 1820)	506.5 (104, 2083)	0.1245
Duration on EFV ≥28 days, n (%)	435 (95.4)	124 (95.4)	311 (95.4)	0.2870
*CYP2B6 516*G>*T* polymorphism, n (%)				<0.0001
*GG*	306 (67.1)	114 (87.7)	192 (58.9)	
*GT*	142 (31.1)	16 (12.3)	126 (38.7)	
*TT*	8 (1.8)	0 (0.0)	8 (2.5)	
Concurrent ART backbone, n (%)				<0.0001
AZT/3TC	49 (10.8)	9 (6.9)	40 (12.3)	
ABC/3TC	88 (19.3)	10 (7.7)	78 (23.9)	
TDF/3TC or TDF/FTC	319 (70.0)	111 (85.4)	208 (63.8)	

*Patients having received combination antiretroviral therapy for at least 180 days were included in the analysis.

**Patients having received combination antiretroviral therapy for between 30 and 179 days were included in the analysis.

***CART-responsiveness was defined by (1) PVL <50 copies/ml for patients who had received cART for at least 180 days; or (2) PVL <50 copies/L or 2 log_10_ decline from baseline to the time of sampling for plasma efavirenz concentrations for patients who had received cART for 30 to 179 days. Patients having received combination antiretroviral therapy for at least 30 days were included in the analysis.

Abbreviations: 3TC, lamivudine; ABC, abacavir; AZT, zidovudine; cART, combination antiretroviral therapy; EFV, efavirenz; FTC, emtricitabine; HBV, hepatitis B virus; HCV, hepatitis C virus; PVL, plasma HIV RNA load; TDF, tenofovir disoproxil fumarate. TDF, tenofovir disoproxil fumarate.

The median plasma EFV C12 was 2.41 mg/L (IQR, 1.93–3.14). The majority of the patients (392/456, 86.0%) had EFV C12 within the proposed therapeutic range of 1 to 4 mg/L, while only 6 (1.3%) had EFV C12 < 1 mg/L (range, 0.352–0.871) and 58 (12.7%) ≥4 mg/L. The samples were taken after a median EFV exposure duration of 473.5 days (IQR, 70–1982) and 21 patients (4.6%) had their samples taken between 2 to 4 weeks after starting EFV-containing cART. CART efficacy did not differ significantly among those with plasma EFV C12 of < 1, 1–4 and ≥4 mg/L (p = 0.658); and all of the 6 patients with plasma EFV C12 <1 mg/L achieved viral suppression.

Among the 34 patients (8.2%) who failed to achieve viral suppression, none had plasma EFV C12 < 1 mg/L. Of the 14 patients whose genotypic resistance testing was performed, resistance-associated mutations to nNRTIs were found in 6 patients (42.9%) with a median plasma EFV C12 of 2.22 mg/L (range, 1.57 to 2.35), which was similar to that of 8 patients (57.1%) in whom no resistance-associated mutations to nNRTIs were detected (2.55 mg/L, [range, 1.53 to 4.15]; p = 0.219).

**Figure 1:**
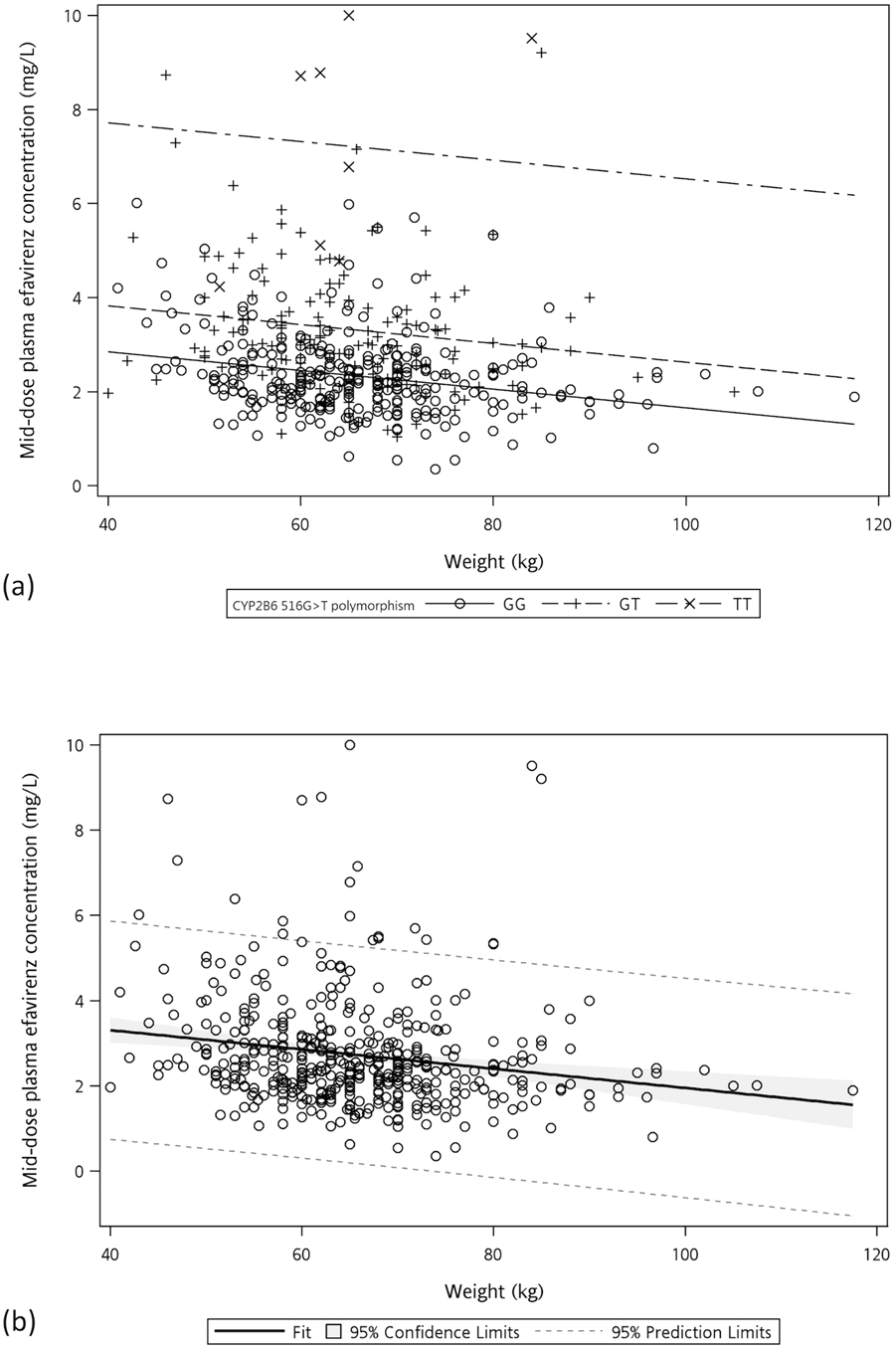
Scattered diagram and linear models for prediction of mid-dose plasma efavirenz concentration. (**a**) The model with CYP2B6 516G>T polymorphism and (**b**) the model without CYP2B6 516G>T polymorphism.

CYP2B6 516G>T polymorphism and weight were identified in the ANCOVA model to have a significant association with plasma EFV C12 (Table 2 and Fig. 1). The duration of EFV exposure was not associated with EFV C12 in the univariate analysis (Table 1 and Supplementary Figure S1). Other variables that had p-values < 0.05 in the univariate analysis, including height, chronic hepatitis B infection, nadir CD4 lymphocyte count, plasma HIV RNA load before cART, CD4 count at the time of determinations of plasma EFV C12, and concurrent ART backbone, were not significantly associated with plasma EFV C12 in the backward selection model.

**Table 2:** Predictors of plasma mid-dose efavirenz concentrations in the analysis of covariance (ANCOVA) models. (a) The model with CYP2B6 516G>T polymorphism and (b) the model without CYP2B6 516G>T polymorphism.

Parameter		Estimate	95% Confidence Limits		p-value
(a)					
Intercept		3.643	3.051	4.236	<0.0001
CYP2B6 516G>T genotype	GG	0			
	GT	+0.976	0.765	1.188	<0.0001
	TT	+4.871	+4.126	+5.616	<0.0001
Weight (per 10-kg increase)		−0.199	−0.287	−0.111	<0.0001
(b)					
Intercept		4.210	3.494	4.926	<0.0001
Weight (per 10-kg increase)		−0.226	−0.334	−0.119	<0.0001

**Figure 2:**
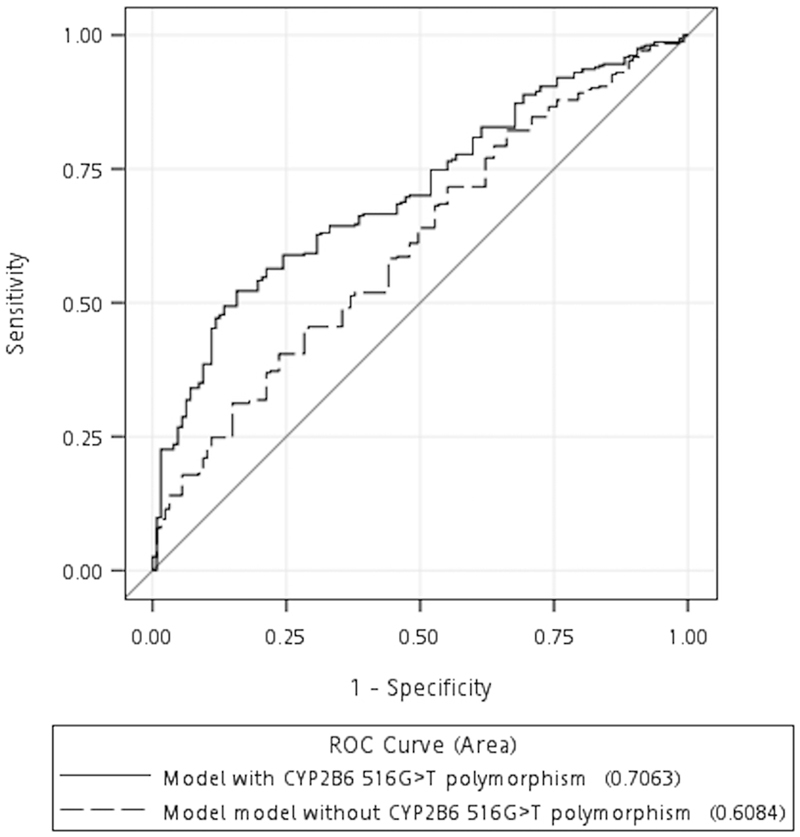
Area-under-the-curves (AUCs) of the receiver-operating-characteristic (ROC) curves of the models predicting plasma mid-dose efavirenz concentration ≥2 mg/L. (Solid line indicating the models with CYP2B6 516G>T genotype and the dashed line indicating the models without CYP2B6 516G>T genotype).

Compared to the GG genotype, the GT and TT genotypes increased the plasma EFV C12 by 0.976 mg/L (95% CI, 0.765–1.188 mg/L) and 4.871 mg/L (95% CI, 4.126–5.616 mg/L), respectively. Meanwhile, a higher weight was associated with decreased EFV C12 by 0.199 mg/L per 10-kg increment (95% CI, 0.111–0.287 mg/L). This prediction model had a moderate predictive value with area under the curve (AUC) of 0.706 (95% CI, 0.656–0.756) for plasma EFV C12 ≥ 2 mg/L (Fig. 2). In the model without CYP2B6 516G>T polymorphism, weight remained a statistically significant predictor of plasma EFV C12 and a 10-kg increase in weight decreased EFV C12 by 0.226 mg/L (95% CI, 0.119–0.334). The AUC decreased to 0.608 (95% CI, 0.551–0.666), representing borderline discrimination. Comparisons of clinical characteristics, distribution of CYP2B6 516G>T genotypes, and plasma EFV C12 among patients of weight groups in quartiles are shown in Supplementary Table S1. The distribution of CYP2B6 516G>T genotypes were similar among the participants in different weight groups (p = 0.112).

In the sensitivity analysis, the backward model selection was performed in different sub-populations including patients with EFV exposure for at least 4 weeks, those for at least 16 weeks and those who were cART-responsive at the time of blood sampling for determinations of plasma EFV C12, all of which yielded similar results (Supplementary Table S2).

**Figure 3:**
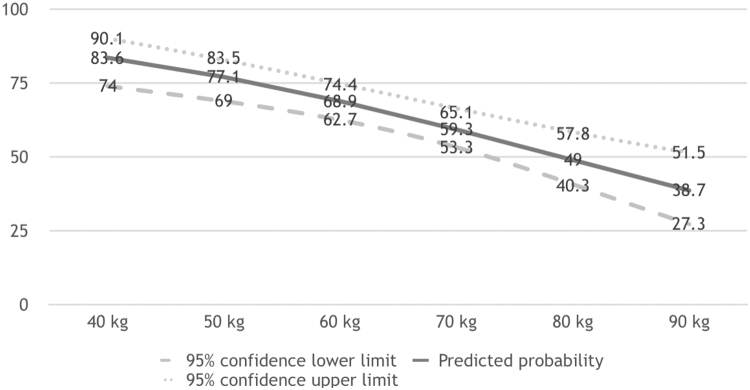
Predicted probability of plasma mid-dose efavirenz concentrations ≥2 mg/L in different weights by binary logistic regression model.

**Figure 4:**
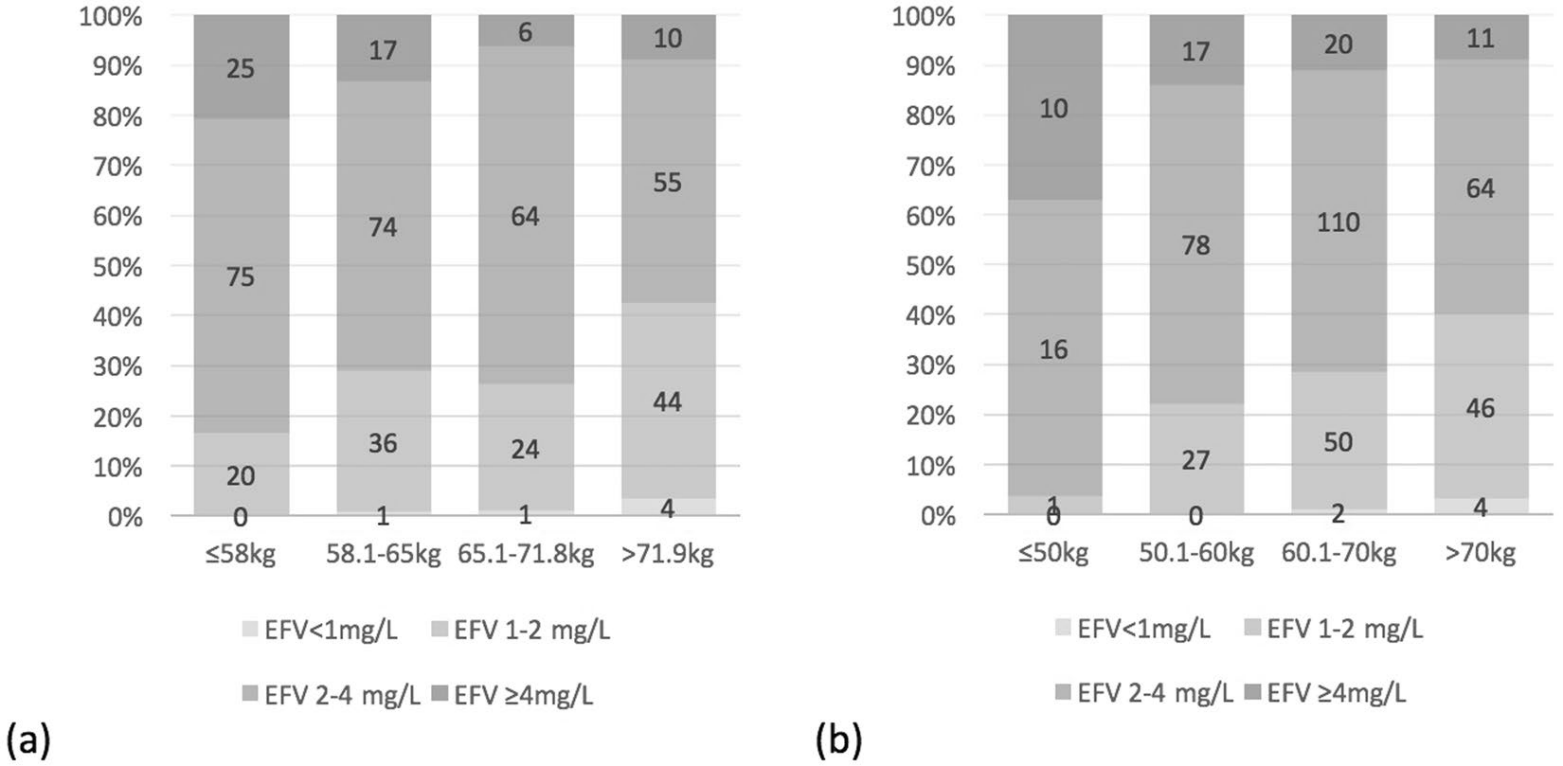
Plasma mid-dose efavirenz concentrations in (**a**) weight quartile groups and (**b**) 10-kg increment groups.

In a *post hoc* analysis, we explored the possibility of using a lower weight ≤50 kg or ≤58 kg (the first quartile) as the predictor of plasma EFV C12 ≥ 2 mg/L. The probabilities of plasma EFV C12 ≥ 2 mg/L were calculated utilizing the model without CYP2B6 516G>T polymorphism in the binary logistic regression analysis (Fig. 3). As a result, patients with a weight of ≤50 kg and ≤58 kg would have a higher predicted probability of 77.1% (95% CI, 69.0 to 83.5) and 70.6% (95%CI, 64.1 to 76.4), respectively, to have plasma EFV C12 ≥ 2 mg/L. Further examinations of our cohort revealed that, among the 120 patients who weighed ≤58 kg, 83.3% and 94.2% had plasma EFV C12 ≥ 2 and ≥1.5 mg/L, respectively; and among the patients who weighed ≤50 kg, only 1 had plasma EFV C12 < 2 mg/L (1.969 mg/L) (Fig. 4). She was a 48-year-old female, had CYP2B6 516GT, and reported irregular dosing of EFV, though her plasma HIV RNA loads had been undetectable over the follow-up period.

## Discussion

Under EFV at a daily dose of 600 mg, more than 98% of the patients in this Taiwanese cohort achieved plasma EFV C12 ≥ 1 mg/L and 71.5% ≥2 mg/L. These findings are similar to those of previous studies that reported that very few (around 3%) patients on standard 600 mg EFV had plasma EFV C12 < 1 mg/L^12,26^. In ENCORE1 study, reducing EFV dose to 400 mg daily achieved the minimum effective concentration of 1 mg/L in more than 95% of the patients, maintained good clinical efficacy, and decreased the risk for drug-related adverse events^9,10,27^. Further analysis from ENCORE1 study showed that among the 20 patients with plasma EFV C12 < 1 mg/L, only 2 failed to achieve suppression of plasma HIV RNA load to <200 copies/mL. This finding, together with ours and others’^25^ suggests that further reduction of EFV dosage could be feasible at least in carefully selected populations.

According to our previous observation, a dose reduction of EFV to 300 mg daily from standard 600 mg daily was associated an average of 46.2% reduction (IQR, 37.7–55.0%) of plasma EFV C12^23^. Therefore, under the guidance of TDM, patients on standard 600 mg EFV with plasma EFV C12 of 2 mg/L or greater may be candidates for dose halving. To identify these individuals, factors associated with plasma EFV C12 were examined in this study. CYP2B6 516G>T genetic polymorphism and body weight were found to be consistently and significantly associated with EFV concentrations. It was previously believed that EFV auto-induction takes 6 to 14 days to complete^28–30^. However, in recent years, it has been documented that this period may be longer, even up to 3 or 4 months^31,32^. The present study enrolled subjects having received EFV for a minimum of 14 days. A small number of these patients might still be in this induction period and their plasma EFV concentrations may not have reached a steady state. To confirm our findings, we excluded patients having received EFV for less than 4 weeks and 16 weeks in the sensitivity analysis. The analyses yielded similar findings (Supplementary Table 2).

EFV is metabolized mainly through CYP2B6 (92%) with a minor contribution from CYP2A6 (7–8%), and UGT2B7 (<1%)^20^. A number of different genetic variations of the cytochrome P450 and uridine 5′-diphospho-glucuronosyltransferase have been reported to influence EFV metabolism, among which CYP2B6 516G>T polymorphism has the strongest predictive value for plasma EFV concentrations^4,17,26^. This genetic variation also explains part of the inter-racial and inter-ethnic differences in plasma EFV concentrations^12,18^. In our study, patients with GT and TT genotypes on CYP2B6 516 locus were predicted to have about 1 mg/L and 5 mg/L higher plasma EFV C12, respectively, compared to those with the wild-type GG genotype. All of the patients with CYP2B6 516TT had plasma EFV C12 ≥ 4 mg/L and about 90% of the patients with non-GG CYP2B6 516G>T genotype had plasma EFV C12 ≥ 2 mg/L. This genetic marker could be a useful tool to predict higher plasma EFV concentrations.

Body weight was inversely related to plasma EFV concentrations in this study as illustrated in Fig. 1. Several previous studies also identified weight or body-mass index as predictors of plasma EFV concentrations in patients with or without concomitant rifampicin^15,16,33,34^. Stohr W *et al*. examined 339 patients from the United Kingdom and reported that the plasma EFV concentrations was 10% lower per additional 10-kg increase of weight. In our study, the patients within the lowest quartile of weight (≤58 kg) had median plasma EFV C12 of 2.80 mg/L and 94.2% of these patients had EFV C12 ≥ 1.5 mg/L. Taking into consideration that the minimum effective concentration has been proposed to be lower than 1 mg/L^24,25^, our findings suggest that a weight of ≤58 kg could be a simple thumb-rule to identify patients with potential for EFV dose reduction in HIV-positive individuals. This observation, if further validated, could be of great benefit in providing safer and more cost-effective treatment to resource-limited areas where HIV-infection and low body weight are common^16,35,36^.

This study has several limitations. First, this is a single-center study including only Taiwanese patients. Studies that include more diverse ethnicities are needed before our findings can be generalized. Of note, Africans have a higher prevalence of CYP2B6 516G>T alleles, and generally have higher plasma EFV concentrations^20^. A practical dose reduction strategy could potentially benefit this population more. Second, this study included only 24 (5.3%) female patients and was underpowered to evaluate the relationship between gender and EFV concentration. In the literature review, some reported significant association between gender and EFV concentration and females generally had higher EFV levels compared to males^12,18,29^; however, others did not reproduce the same findings^8,15,26,32,33^. As EFV is part of the recommended first-line treatment for all adult women, studies that involve more women are needed urgently before the findings of this study can be applied to them. Third, although patients’ self-reporting of good compliance was required before enrollment in this study, we did not measure adherence objectively. Without controlling for this factor, potential confounders probably existed and may have decreased the predictive value of our models. Fourth, several genetic variations other than CYP2B6 516G>T polymorphism, including CYP2B6 983 T>C, CYP2B6 785 A>G, CYP2A6*9 and CYP2A6*17, have been documented to have an impact on plasma EFV concentrations^20^ and these factors were not examined in our study. While including all these pharmacogenetic variables would potentially improve the predictive value of the model, this improvement is unlikely to be translated into an additional clinical benefit in most real world settings as these tests are not widely available. Last, data of resistance-associated mutations to efavirenz among the patients with inadequate treatment responses were collected retrospectively. While the association between EFV concentrations and emergence of resistance-associated mutations were not found, this should be interpreted with caution as the number of patients undergoing genotypic resistance testing remains small.

In conclusion, in this study involving HIV-positive patients on 600 mg EFV in Taiwan, the majority had plasma EFV C12 > 1 mg/L. CYP2B6 516G>T polymorphism and body weight were associated with higher plasma EFV C12. While more clinical studies are needed to confirm the effectiveness of EFV dose that is lower than 400 mg daily, non-GG genotype of CYP2B6 516G>T locus and body weight <58 kg were predictive of plasma EFV C12 ≥ 2 mg/L and could be used to identify potential candidates for EFV dose reduction to 300 mg daily.

## Materials and Methods

### Study Population

From October 2009 to July 2016, HIV-positive patients aged 18 years or older who had received EFV at a daily dose of 600 mg for a minimum of 14 days were enrolled when they sought routine HIV care at the National Taiwan University Hospital, the largest referral hospital for inpatient and outpatient HIV care in Taiwan. Patients were excluded if they were pregnant or receiving concomitant anti-tuberculous agents, anti-epileptics or protease inhibitors; if they had questionable adherence to their antiretroviral therapy; or if they were critically ill. The protocol was approved by the Research Ethics Committee of the National Taiwan University Hospital (registration number, 200908014 M) and all patients provided written informed consent prior to enrollment. The study was carried out in accordance with the approved ethical guidelines and regulations.

### Data Collection and Sample Preparation

A computerized case record form was used to collect data on the demographics, weight and height, concomitant medications, duration of cART and EFV exposure, serostatus of hepatitis B virus (HBV) and hepatitis C virus (HCV), serum liver enzymes and creatinine levels, CD4 lymphocyte counts and plasma HIV RNA loads (before the initiation of cART and within 2 weeks from the time of pharmacokinetic study), and the patient’s self-reported date and time of the last EFV dose. After enrollment, blood samples were collected 12 ± 2 hours after the last dose of EFV with tubes containing EDTA as anticoagulant for CYP2B6 516G>T genotyping and determinations of plasma EFV C12. Plasma samples were stored at −80 °C until analysis.

### Laboratory Investigations

Plasma HIV RNA load was quantified using Cobas Ampliprep HIV-1 test (Cobas Ampliprep version 1.0 before June 2013 and version 2.0 afterward, Roche Diagnostics Corporation, IN) and the detection limits changed from 40 to 20 copies/mL since June 2013. CD4 lymphocyte count was determined using FACFlow (BD FACS Calibur, Becton Dickinson, CA). The CD4 counts and plasma HIV RNA loads were monitored 28 to 30 days after initiation of cART in antiretroviral-naïve patients, before change of regimens in the presence of virological failure, and every 3 to 6 months thereafter according to the national HIV treatment guidelines. Transmitted drug resistance mutations of HIV-1 to NRTIs, nNRTIs, protease inhibitors, and integrase inhibitors were not routinely determined before cART was initiated; genotypic resistance testing was only performed retrospectively for the purposes of surveillance^37,38^.

High molecular weight genomic DNA was extracted from PBMC using the Wizard® Genomic DNA purification kit (Promega, WI, USA). The concentration of extracted DNA was determined by spectrophotometry and stored at −20 °C before further analysis. Polymerase-chain-reaction restriction fragment-length polymorphism (PCR-RFLP) was performed to determine the single-nucleotide polymorphism (SNP) of CYP2B6 516G>T.

Plasma concentrations of EFV were quantified using a validated high-performance liquid chromatography (HPLC) assay with UV detection at a wavelength of 245 nm. Analysis of independently prepared quality control samples indicated good reproducibility (coefficients of variation ≤0.27%) and accuracy (measured concentrations <±3% from target concentrations). The calibration curve was linear within the range of 0.5 to 10.0 mg/L^17^.

### Statistical Analysis

Statistical analysis was performed using the SAS software (version 9.4). The plasma HIV RNA load values were log-transformed with those values below the detection limits set as 39 copies/mL. We defined ART-responsiveness by plasma HIV RNA load <50 copies/mL for those who had been on cART for more than 6 months at the time of enrollment; and, for those who initiated cART for less than 6 months, cART-responsiveness was defined by plasma HIV RNA load <50 copies/ml or a 2-fold log decline of the plasma HIV RNA load from baseline. Elevated serum transaminase was defined when either serum alanine transaminase (ALT) or aspartate transaminase (AST) exceeded upper limit of normal reference range (41 IU/L for ALT and 31 IU/L for AST). Estimated glomerular filtration rate (eGFR) was calculated using simplified modification of diet in renal disease (MDRD) formula. Categorical variables were expressed as numbers and percentages, while continuous variables were reported as medians and interquartile ranges (IQR).

Variables including age, gender, weight, height, concomitant ART backbone, duration on cART and EFV, serostatus of HBV and HCV, abnormalities in transaminases and eGFR, CD4 lymphocyte counts, plasma HIV RNA loads, and CYP2B6 516G>T polymorphism were entered into a backward model selection with dependent variable being plasma EFV C12. The independent predictors included in analysis of covariance (ANCOVA) model were identified (model with CYP2B6 516G>T polymorphism). A second model was constructed under the same analytic procedures but with exclusion of CYP2B6 516G>T polymorphism (model without CYP2B6 516G>T polymorphism). We defined plasma EFV C12 of 2 mg/L as a cut-off value for identifying the potential patients for EFV dose reduction to 300 mg daily^23^. The predictors were then entered into binary logistic regression model with outcome set to be plasma EFV C12 ≥ 2 mg/L. Area-under-the-curves (AUCs) of the receiver-operating-characteristic (ROC) curves were calculated to measure the predictive value of these models.

The proposed models were further tested in patients who were cART-responsive and in patients who had received EFV for at least 4 weeks and those for at least 16 weeks at the time of blood sampling for determinations of plasma C12 as a measure of sensitivity analysis. The variables with p-value < 0.05 were deemed statistically significant throughout the analyses.

### Availability of materials and data

The datasets generated during and/or analysed during the current study are available from the corresponding author on reasonable request.

## Electronic supplementary material

Supplementary tables and figure
