# Supplementary material for: Prediction of plasma efavirenz concentrations among HIV-positive patients taking efavirenz-containing combination antiretroviral therapy
Source: Sci Rep. 2017 Nov 23;7:16187. doi: 10.1038/s41598-017-16483-2 (PMC5701031; doi:10.1038/s41598-017-16483-2)

**Prediction of plasma efavirenz concentrations among HIV-positive patients taking efavirenz-containing combination antiretroviral therapy**

Sung-Hsi Huang^1^, Shu-Wen Lin^2,3,4^, Sui-Yuan Chang^5,6^, Ya-Ting Lin^3^, Chieh Chiang^7^, Chin-Fu Hsiao^7^, Hsin-Yun Sun^8^, Wen-Chun Liu^8^, Yi-Ching Su^8^, Chien-Ching Hung^8,9,10,11,*^, Shan-Chwen Chang^3,8^

**Supplementary table S1**. Comparisons of clinical characteristics, CYP2B6 516G>T genotypes, and plasma mid-dose efavirenz concentrations among the patients of weight quartile groups.

|  | **Weight** | | | | **p-value** |
| --- | --- | --- | --- | --- | --- |
|  | ≤58  (N=120) | 58.1-65.0  (N=128) | 65.1-71.8  (N=95) | ≥71.9  (N=113) |  |
| **Age, median (IQR), years** | 35.0 (28.9, 42.8) | 37.7 (29.5, 45.7) | 35.6 (29.3, 42.3) | 35.7 (29.8, 41.5) | 0.770 |
| **Male sex, n (%)** | 101 (84.2) | 125 (97.7) | 94 (98.9) | 112 (99.1) | <0.001 |
| **Mid-dose EFV concentration, median (IQR), mg/L** | 2.80 (2.19, 3.65) | 2.37 (1.91, 3.13) | 2.33 (1.98, 2.89) | 2.13 (1.78, 2.79) | <0.001 |
| **Chronic HBV infection, n (%)** | 20 (16.7) | 19 (14.8) | 18 (18.9) | 18 (15.9) | 0.865 |
| **Chronic HCV infection, n (%)** | 8 (6.7) | 9 (7.0) | 6 (6.3) | 7 (6.2) | >0.999 |
| **Elevated aminotransferase, n (%)** | 18 (15.0) | 14 (10.9) | 14 (14.7) | 29 (25.7) | 0.022 |
| **Estimated glomerular filtration rate <90 ml/min/1.73 m^2^, n (%), (N=455)** | 22 (18.3) | 29 (22.7) | 26 (27.4) | 25 (22.3) | 0.474 |
| **Nadir CD4, median (IQR), cells/mm^3^ (N=441)** | 217 (77, 346) | 247.5 (92, 363.5) | 231 (82, 386) | 217 (63, 393) | 0.850 |
| **Nadir CD4 <200 cells/mm^3^, n (%) (N=441)** | 51 (44.3) | 49 (39.5) | 42 (45.2) | 50 (45.9) | 0.756 |
| **PVL before initiation of cART, median (IQR), log_10_ copies/ml (n=424)** | 4.85 (4.54, 5.42) | 4.95 (4.48, 5.46) | 4.89 (4.49, 5.46) | 4.80 (4.32, 5.38) | 0.621 |
| **CD4 at the time of sampling for plasma efavirenz concentrations, median (IQR), cells/mm^3^ (N=450)** | 527  (373, 713) | 492  (358, 605) | 508  (361, 666.5) | 483  (334, 682) | 0.269 |
| **PVL at the time of sampling for plasma efavirenz concentrations, median (IQR), log_10_ copies/ml (N=450)** | 1.59  (1.59, 1.88) | 1.59  (1.59, 1.84) | 1.59  (1.59, 1.61) | 1.59  (1.59, 1.69) | 0.656 |
| **PVL <50 copies/ml at the time of sampling for plasma efavirenz concentrations, n (%) (N=304)*** | 73 (96.1) | 79 (95.2) | 67 (95.7) | 68 (90.7) | 0.515 |
| **PVL <50 copies/ml or 2 log_10_ decline from baseline to the time of sampling for plasma efavirenz concentrations, n (%) (N=415)**** | 102 (97.1) | 114 (95.0) | 80 (94.1) | 99 (94.3) | 0.700 |
| **Duration on cART, median (IQR), days** | 575 (42, 3525) | 439 (96, 1997) | 698 (135, 2149) | 365 (70,1965) | 0.492 |
| **On EFV as the first line treatment, n (%)** | 95 (79.2) | 111 (86.7) | 80 (84.2) | 102 (90.3) | 0.116 |
| **Duration on EFV, median (IQR), days** | 564 (42, 3162) | 427 (91, 1919) | 604 (117, 1953) | 307 (66, 1416) | 0.426 |
| **Duration on EFV ≥28 days, n (%)** | 114 (95.0) | 123 (96.1) | 87 (91.6) | 110 (97.3) | 0.278 |
| ***CYP2B6 516G>T* polymorphism, n (%)** |  |  |  |  | 0.112 |
| ***GG*** | 75 (62.5) | 87 (68.0) | 69 (72.6) | 75 (66.4) |  |
| ***GT*** | 44 (36.7) | 35 (27.3) | 26 (27.4) | 37 (32.7) |  |
| ***TT*** | 1 (0.8) | 6 (4.7) | 0 (0.0) | 1 (0.9) |  |
| **Concurrent cART backbone, n (%)** |  |  |  |  | 0.749 |
| **Coformulated AZT/3TC** | 25 (20.8) | 28 (21.9) | 15 (15.8) | 20 (17.7) |  |
| **Coformulated ABC/3TC** | 15 (12.5) | 13 (10.2) | 12 (12.6) | 9 (8.0) |  |
| **TDF plus 3TC or coformulated TDF/FTC** | 80 (66.7) | 87 (68.0) | 68 (71.6) | 84 (74.3) |  |

*Patients having received combination antiretroviral therapy for at least 180 days were included in the analysis.

**Patients having received combination antiretroviral therapy for at least 30 days were included in the analysis.

Abbreviations: 3TC, lamivudine; ABC, abacavir; AZT, zidovudine; cART, combination antiretroviral therapy; EFV, efavirenz; FTC, emtricitabine; HBV, hepatitis B virus; HCV, hepatitis C virus; PVL, plasma HIV RNA load; TDF, tenofovir disoproxil fumarate

**Supplementary table S2**. Predictors of plasma mid-dose efavirenz concentrations in the analysis of covariance (ANCOVA) models. (a) The model selected from patients having received efavirenz for at least 4 weeks; (b) the model selected from patients having received efavirenz for at least 16 weeks; (c) the model selected from patients with positive virological response to combination antiretroviral therapy.

(a)

| **Parameter** |  | **Estimate** | **95% Confidence Limits** | | **p-value** |
| --- | --- | --- | --- | --- | --- |
| Intercept |  | 3.725 | 3.136 | 4.313 | <0.0001 |
| CYP2B6 516G>T genotype | GG | 0 |  |  |  |
|  | GT | +0.904 | 0.693 | 1.115 | <0.0001 |
|  | TT | +4.464 | +3.706 | +5.223 | <0.0001 |
| Weight (per 10-kg increase) |  | -0.210 | -0.298 | -0.123 | <0.0001 |

(b)

| **Parameter** |  | **Estimate** | **95% Confidence Limits** | | **p-value** |
| --- | --- | --- | --- | --- | --- |
| Intercept |  | 3.789 | 3.075 | 4.503 | <0.0001 |
| CYP2B6 516G>T genotype | GG | 0 |  |  |  |
|  | GT | +0.982 | 0.737 | 1.226 | <0.0001 |
|  | TT | +4.634 | +3.716 | +5.553 | <0.0001 |
| Weight (per 10-kg increase) |  | -0.213 | -0.319 | -0.106 | 0.0001 |

(c)

| **Parameter** |  | **Estimate** | **95% Confidence Limits** | | **p-value** |
| --- | --- | --- | --- | --- | --- |
| Intercept |  | 3.712 | 3.102 | 4.322 | <0.0001 |
| CYP2B6 516G>T genotype | GG | 0 |  |  |  |
|  | GT | +0.987 | 0.767 | 1.207 | <0.0001 |
|  | TT | +5.343 | +4.552 | +6.135 | <0.0001 |
| Weight (per 10-kg increase) |  | -0.211 | -0.301 | -0.120 | <0.0001 |

**Supplementary figure S1**. Mid-dose efavirenz concentration in patients with (a) efavirenz exposure of <42 days, 42 to 179 days, and ≥180 days and (b) efavirenz exposure of <30 days, 30 to 89 days, and 90 to 180 days. The efavirenz concentrations were similar among groups with different durations of efavirenz exposure (*p* = 0.1892 and *p* = 0.0844, respectively).

(a)
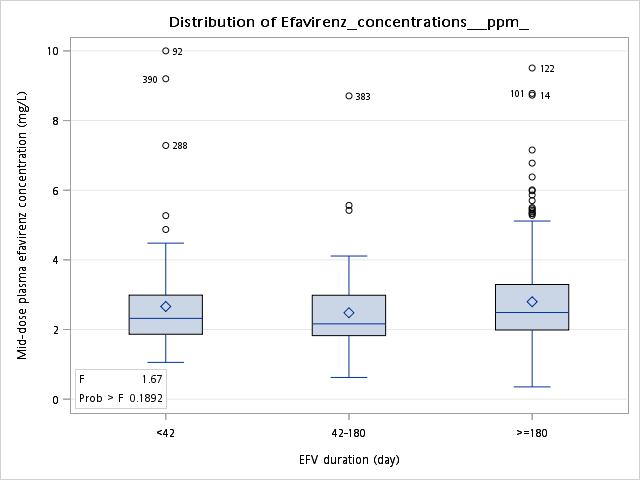


(b)
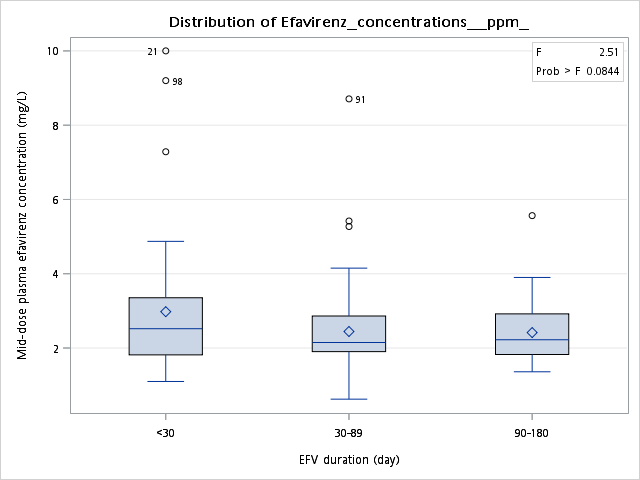

Supplement: Supplementary file 1 — Supplementary tables and figure [file 41598_2017_16483_MOESM1_ESM.doc]
